# A Paradigm Shift: Rehabilitation Robotics, Cognitive Skills Training, and Function After Stroke

**DOI:** 10.3389/fneur.2019.01088

**Published:** 2019-10-15

**Authors:** Susan E. Fasoli, Catherine P. Adans-Dester

**Affiliations:** ^1^Department of Occupational Therapy, School of Health and Rehabilitation Sciences, Massachusetts General Hospital Institute of Health Professions, Boston, MA, United States; ^2^Department of Physical Medicine and Rehabilitation, Harvard Medical School, Spaulding Rehabilitation Hospital, Boston, MA, United States

**Keywords:** stroke, robot-assisted therapy (RAT), upper extremity (UE), cognitive strategy training, activity performance, transfer of training strategies, motor learning

## Abstract

**Introduction:** Robot-assisted therapy for upper extremity (UE) impairments post-stroke has yielded modest gains in motor capacity and little evidence of improved UE performance during activities of daily living. A paradigm shift that embodies principles of motor learning and exercise dependent neuroplasticity may improve robot therapy outcomes by incorporating active problem solving, salience of trained tasks, and strategies to facilitate the transfer of acquired motor skills to use of the paretic arm and hand during everyday activities.

**Objective:** To pilot and test the feasibility of a novel therapy protocol, the Active Learning Program for Stroke (ALPS), designed to complement repetitive, robot-assisted therapy for the paretic UE. Key ALPS ingredients included training in the use of cognitive strategies (e.g., STOP, THINK, DO, CHECK) and a goal-directed home action plan (HAP) to facilitate UE self-management and skill transfer.

**Methods:** Ten participants with moderate impairments in UE function >6 months after stroke received eighteen 1-h treatment sessions 2–3/x week over 6–8 weeks. In addition to ALPS training, individuals were randomly assigned to either robot-assisted therapy (RT) or robot therapy and task-oriented training (RT-TOT) to trial whether the inclusion of TOT reinforced participants' understanding and implementation of ALPS strategies.

**Results:** Statistically significant group differences were found for the upper limb subtest of the Fugl-Meyer Assessment (FMA-UE) at discharge and one-month follow-up favoring the RT group. Analyses to examine overall effects of the ALPS protocol in addition to RT and RT-TOT showed significant and moderate to large effects on the FMA-UE, Motor Activity Log, Wolf Motor Function Test, and hand portion of the Stroke Impact Scale.

**Conclusion:** The ALPS protocol was the first to extend cognitive strategy training to robot-assisted therapy. The intervention in this development of concept pilot trial was feasible and well-tolerated, with good potential to optimize paretic UE performance following robot-assisted therapy.

## Introduction

Rehabilitation efforts to optimize motor function, activity performance and participation after stroke require an understanding of factors that contribute to stroke recovery and an intervention approach focused on the individual's goals and desire to re-engage in valued life roles. Despite recent advances in acute medical interventions to reduce the impact of stroke, residual upper extremity (UE) motor deficits persist long term in up to 65% of stroke survivors, contributing to a loss of independence in activities of daily living and negatively impacting quality of life (1). To advance rehabilitative practice and facilitate satisfaction and participation after stroke, improved methods are needed to optimize the recovery of motor function for home and community activities.

Evidence of neural recovery following highly intensive therapy and the high cost of health care have driven the development of rehabilitation robots to treat motor impairments after stroke. Rehabilitation robots have provided researchers and clinicians with new treatment options to improve UE motor capacity and performance after stroke. The number of robot-assisted therapy trials to address UE function has grown significantly over the past 20 years. Previous studies have shown robot-assisted therapy to be as effective as repetitive task-specific training at increasing motor capacity, as measured by standard assessments in clinical settings (2, 3). While systematic reviews of robot-assisted therapies confirm gains in motor capacity after stroke, they provide little evidence for the transfer of trained motor skills to paretic UE performance during activities of daily living (4, 5). This disparity between improved UE motor capacity (i.e., what a person can do in a standardized, controlled setting) and daily use of the paretic arm and hand is a significant clinical issue (6) and critical barrier to the integration of robotic technology into clinical practice. These findings may be attributed to the limited development of rehabilitation robots that specifically train voluntary control of finger flexion and extension of the paretic hand, and a primary focus on intensity of practice with little regard for other *principles of motor learning and experience-dependent neuroplasticity* (7, 8). These principles, including the salience of training tasks, transfer of acquired skills to similar activities, and active engagement and problem solving, are key to task-oriented training paradigms in stroke but have not been well-integrated into robot-assisted therapy protocols. Recent studies on the use of active problem solving and guided discovery to facilitate skill acquisition during task-oriented training have demonstrated transfer to untrained tasks (9) and significant improvements on measures of UE motor capacity and performance after stroke (10). While these treatment components are instrumental to the transfer of motor skills acquired during task-oriented training, they previously have been absent in robot-assisted therapy trials.

### Objectives

The primary aim of this pilot study was to develop and refine a theory-based stroke therapy protocol, the Active Learning Program for Stroke (ALPS), to facilitate the transfer of robot-trained UE motor skills to functional use of the paretic arm and hand during every day activities. The secondary aim was to examine effects of ALPS training combined with either robot-assisted therapy or robot therapy + task-oriented training. We hypothesized that the intervention would be feasible and well-tolerated by participants and would yield positive outcomes on standard measures of paretic UE motor capacity and performance across domains of the International Classification of Functioning, Disability and Health (ICF) (11). This study has potential for improving the effectiveness of robot-assisted therapy by facilitating UE self-management and specifically addressing the transfer of acquired skills (e.g., UE motor capacity) to the performance of UE tasks during activities of daily living. The ALPS protocol is relevant to clinical practice because it provides clinicians with a structured, client-centered motor learning approach to optimize use of the paretic arm and hand.

### Active Learning Program for Stroke (ALPS): Conceptual Framework and Application

The ALPS protocol is based upon principles of experience dependent neuroplasticity as described by Kleim and Jones (7); empirical evidence from UE motor learning and task-oriented training programs for individuals with stroke (8, 12); and a conceptual framework for integrating skill, capacity and motivation as described in multiple publications by Winstein et al. (12–14). While principles of repetition, intensity, and specificity of training are active ingredients of robot-assisted therapy protocols to improve motor capacity, other motor learning principles, such as salience and transference, have not been well-infused into prior robot training programs. The ALPS protocol incorporates these principles during robot-assisted therapy sessions, and they are an integral component of each participant's home action plan (HAP) aimed to facilitate UE performance in the home and community. Examples of learning principles are highlighted in Table 1.

**Table 1:** ALPS motor learning principles.

**Motor learning principles**	**Example**
Use it or lose it (7, 13)	Identify interfering and changeable impairments
	Provide targeted UE training based on individual's motor capacity
Salience of training tasks (7)	Establish clear patient-centered goals
Transference (7, 8, 12)	Facilitate UE self-management through active problem identification and problem solving
Feedback (9, 10, 15)	Provide knowledge of performance
	Encourage self-assessment and discovery
Motivation (7, 8)	Assure challenging and meaningful practice
	Address self-efficacy and confidence

The ALPS protocol involves instructions to engage in active problem solving, activity analysis and use of general cognitive strategies (e.g., STOP, THINK, DO, CHECK), modeled after the Cognitive Orientation for daily Occupational Performance (CO-OP) (15), during paretic UE tasks. We purposely altered our strategy approach from that used in CO-OP because we found that individuals typically don't explicitly establish goals for performance prior to activity engagement. Rather, when they run into challenges while attempting to use their paretic UE functionally they benefit from cues to stop and identify factors impeding performance. Examples of general and domain specific movement strategies are shown in Appendix A.

In conjunction with cognitive strategy training, individuals are provided with a HAP to encourage the application of ALPS principles and use of the paretic UE when engaged in everyday activities in the home and community. Participants identify specific, achievable tasks for their HAP based on personal interests. The clinician may use scores from the upper limb subtest of the Fugl-Meyer Assessment (FMA-UE) (16, 17) when providing input to select appropriate tasks based on the participant's current level of function. Due to this participant-centered approach, there are no core tasks included in every HAP, however, similarities do occur across individuals. Participants identify 3–5 UE tasks to be completed daily at home and are taught general and specific ALPS strategies that may facilitate performance. Participants are encouraged to engage in HAP tasks for at least 30 min each day.

## Materials and Methods

### Study Design

**Figure 1:**
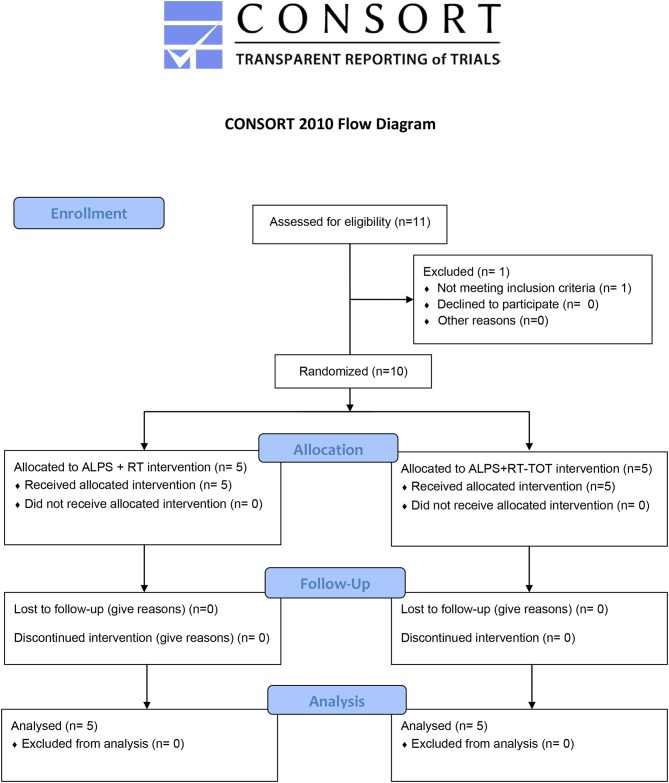
CONSORT flow diagram.

While the primary aim was to develop and refine the ALPS protocol for use with robot-assisted therapy, we were also interested in learning whether the inclusion the both robot-assisted therapy and task-oriented training during treatment sessions reinforced participants' understanding and implementation of ALPS strategies. This single-blind randomized control pilot study examined effects of the ALPS protocol combined with robot-assisted therapy alone, or robot-assisted therapy plus task-oriented training, as described below. The clinical evaluator was blinded to group assignment and research hypotheses (Figure 1).

### Recruitment

Individuals between the ages of 18–82 years and diagnosed with stroke more than 6 months prior to study enrollment were recruited for this study. Informational flyers were provided to attending physicians, outpatient therapists and stroke survivors who previously had given permission to be contacted about research opportunities at Spaulding Rehabilitation Hospital, Boston MA. Inclusion criteria were: moderate UE hemiparesis with initial score on the upper limb subtest of the Fugl-Meyer Assessment (FMA-UE) between 21 and 50/66) (18); and intact cognitive function to understand and actively engage in the ALPS protocol as measured by a Montreal Cognitive Assessment Score of ≥26/30 (19) during the initial evaluation visit. Exclusion criteria were: no more than moderate impairments in paretic UE sensation, passive range of motion, and pain as assessed with the Fugl-Meyer Assessment (18); increased muscle tone as indicated by score of ≥3 on the Modified Ashworth Scale (20); hemispatial neglect or visual field loss measured by the symbol cancellation subtest on the Cognitive Linguistic Quick Test (21); and aphasia sufficient to limit comprehension and completion of the treatment protocol. Participants could not be enrolled in other UE therapy or research during the study period or present with contraindications for robot-assisted therapy, including recent fracture or skin lesion of paretic UE.

The study protocol was reviewed and approved by the Partners Human Research Committee, the Institutional Review Board for Partners HealthCare, and registered at https://clinicaltrials.gov (NCT02747433). All participants provided written informed consent in accordance with the Declaration of Helsinki.

### Intervention

All enrolled participants were administered the ALPS protocol and were randomly assigned to one of two treatment groups: (1) Robot-Assisted Therapy (ALPS + RT) or (2) Robot-Assisted Therapy + Task-Oriented Training (ALPS + RT-TOT).

#### Robot-Assisted Therapy (RT)

**Figure 2:**
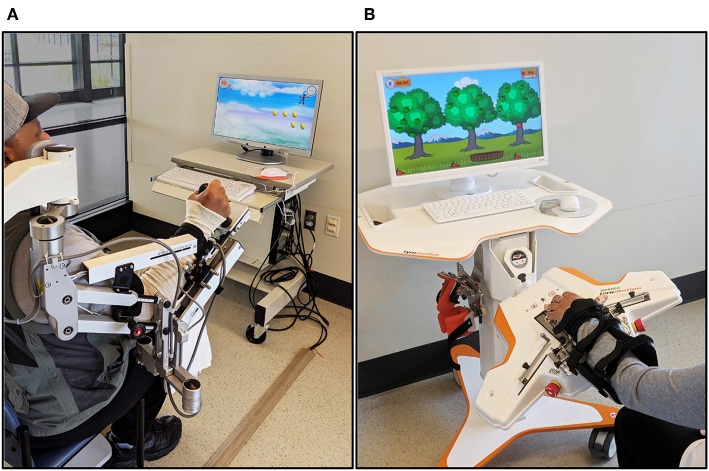
Rehabilitation robots. **(A)** Armeo®Spring **(B)** Amadeo™.

Participants received robot-assisted UE therapy using two commercially-available rehabilitation devices: the Armeo®Spring (Hocoma AG, Switzerland) and Amadeo™ (Tyromotion, Graz, AT) (Figure 2).

The Armeo®Spring is a passive exoskeletal spring suspension system that provides repetitive practice of virtual goal-directed reaching tasks for the paretic UE. A distal sensor that detects grip pressure allows the grasp and release of virtual objects during computer-generated games. The amount of gravity assistance and virtual task demands are selected by the clinician to provide challenging yet achievable movement therapy.

During the first treatment session, the Armeo®Spring was adjusted for the participant's arm size and required angle of suspension (~45° shoulder flexion, 25° elbow flexion) and the workspace was measured via standard device operation procedures. The versatility of the Armeo®Spring system allowed repetitive practice of single degree-of-freedom motions (e.g., elbow flexion/extension, supination/pronation) as well as multiple degree-of-freedom training for the paretic shoulder, elbow, forearm, wrist, and hand.

The Amadeo™ robotic system provides position-controlled exercises during computerized games that emphasize grasp and release of the paretic hand. Participants were seated comfortably with the paretic forearm and wrist strapped to an adjustable support attached to the robot device with the wrist in approximately neutral position. A small magnetic disc was secured to the distal phalanx of each digit for connection to the robotically controlled slide that guides movement. Each 1-h session included visually evoked games that provided active-assistive training of collective and individual flexion and extension of the digits, isometric flexion/extension contractions, and continuous passive motion with visual feedback to rest and relax digits when fatigue or increased muscle tone began to impact motor performance.

All participants received 1-h sessions, 2–3×/week for 6–8 weeks (total 18 sessions), divided into two 9 session treatment blocks. The two treatment blocks were given in order, with all participants receiving proximal training via Armeo®Spring during the first block followed by Amadeo™ distal training during the second block. All training sessions for one treatment block were completed before proceeding to the next. The robot training sessions provided highly repetitive movement training, and the robot training time completed during each session was recorded. Rest periods were offered between computer-generated games, as needed. Task challenge for each training device was incrementally increased or decreased based on participant performance.

#### Task-Oriented Training (TOT)

Participants randomized to the robot and task-oriented training (RT-TOT) group received therapist-guided task-oriented training in addition to RT during 20–30 min of each 1-h treatment session. The participant's baseline performance on the FMA was reviewed, and the FMA keyform and patient-targeted treatment activities outlined by Woodbury et al. (17) aided the selection of UE tasks with greatest potential for improvement during TOT. While we tracked the number of repetitions performed and/or time that participants engaged in continuous motions (e.g., wiping table) the actual dose of TOT differed among participants, based on their activity tolerance and level of function. We attempted to control for this difference by assuring that the overall treatment dose (duration and frequency of therapy sessions) was comparable across RT and RT-TOT groups.

#### ALPS Protocol

Participants randomly assigned to both intervention groups (RT and RT-TOT) received ALPS cognitive strategy training (e.g., STOP, THINK, DO, CHECK), as described above, during each treatment session. The UE training during RT and RT-TOT reinforced the importance of repetitive practice to optimize motor capacity and performance. Guided discovery during RT facilitated participant understanding of how robot-trained motor skills could generalize to everyday tasks. Individuals randomized to the RT-TOT group also engaged in dynamic performance analysis to identify breakdowns in task completion and attempt solutions during “real-life” activities, such as retrieving objects from the fridge (15). Clinician feedback encouraged self-assessment and knowledge of performance, and participants were motivated to explore ways to use their paretic UE better for HAP tasks. Level of engagement, strategy use, achievements, and concerns regarding the completion of the HAP were reviewed at each session. Participants engaged in active problem solving to identify specific strategies to facilitate success by modifying motor actions (e.g., changing body position, assisting with the less affected UE) or activity demands. The HAP was updated weekly to include new everyday activities and strategies to optimize performance and transfer of motor skills trained during robot therapy.

### Outcomes

Clinical assessments were administered at baseline, discharge (<1 week after intervention), and at a 1-month follow-up visit. Evaluation sessions lasted ~1 ½ to 2 h, and the standardized measures listed in Table 2 were administered. All are reliable and valid measures of UE motor function, activity performance and participation for individuals post-stroke.

**Table 2:** Outcome measures.

**ICF DOMAIN/ASSESSMENTS**
**Body Functions**
Fugl-Meyer Assessment—UE (FMA-UE), pain, sensation subtests (18)
Modified Ashworth Scale (MAS) (20)
**Activities and Participation**
Wolf Motor Function Test (WMFT) (22)
Motor Activity Log (MAL) (23)
Stroke Impact Scale (SIS) (24)
Confidence in Arm & Hand Movement (CAHM) (Lewthwaite et al., unpublished)

### Statistical Analysis

We first performed non-parametric Mann Whitney *U* tests to examine effects of ALPS training combined with RT vs. RT-TOT from admission to discharge, and from admission to the 1-month follow-up assessment. To determine whether the addition of ALPS training to RT and RT-TOT resulted in significant gains on measures across ICF domains, raw scores from both groups were combined and Friedman tests examined whether changes in performance at these three time points were significant. *Post-hoc* analyses with Wilcoxon signed-rank tests were conducted. In addition, Cohen's *d* effect sizes for dependent samples were calculated in Microsoft Excel for Office 365. Analyses were completed with the IBM SPSS, Version 25.0 Statistical Package.

## Results

Ten individuals (53.19 ± 19.83 years of age) more than 6 months post-stroke onset participated in this study between July 2016 and November 2018. Participant characteristics for each group are reported in Table 3. Group differences in baseline demographics and FMA-UE scores were non-significant.

**Table 3:** Participant baseline characteristics.

	**RT** **(*n* = 5)**	**RT-TOT** **(*n* = 5)**	**Total** **(*n* = 10)**	***p***
**Age**				
Years, mean ± SD	59.86 ± 19.81	46.51 ± 19.52	53.19 ± 19.83	0.31
**Gender**				
Female/male, *n* (%)	1 (20)/4 (80)	3 (60)/2 (40)	4 (40)/6 (60)	0.19
**Time since stroke**				
Months, mean ± SD	19.91 ± 22.28	97.60 ± 84.06	58.75 ± 70.98	0.11
**Hemiparesis**				
Left/right, *n* (%)	3 (60)/2 (40)	2 (40)/3 (60)	5 (50)/5 (50)	0.52
**Affected side**				
Dominant/non-dominant, *n* (%)	2 (40)/3 (60)	4 (80)/1 (20)	6 (60)/4 (40)	0.47
**Fugl-Meyer assessment upper extremity**				
Score (/66), mean ± SD	34.40 ± 6.73	34.00 ± 12.41	32.20 ± 9.60	0.59

The ALPS protocol was feasible and well-tolerated, as participants (*n* = 10) completed all assessment and intervention sessions, described use of ALPS cognitive strategies during their HAPs, and reported high satisfaction with the therapy process.

Mann Whitney *U* tests revealed statistically significant gains on the FMA-UE from admission to discharge (*Z* = −2.32, *p* = 0.02) and admission to the 1-month follow-up assessment (*Z* = −2.64, *p* = 0.008), with the RT group outperforming those who received RT-TOT. No between-group differences were found for the remaining clinical outcome measures following intervention. Friedman tests and *post-hoc* Wilcoxon analyses to evaluate effects of the ALPS protocol in addition to RT and RT-TOT (*n* = 10) revealed statistically significant improvements at discharge and follow-up for the FMA-UE, WMFT, MAL (AOU and HW scales), and the hand portion of the SIS (see Table 4).

**Table 4:** Friedman analyses (*n* = 10).

**Outcome measure**	**Baseline**	**Post- intervention**	**1-month follow-up**	**Significance**
**FMA-UE (0-66)**				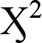 (2) = 13.26^***^
Mean	32.20	39.50	39.50	*p* = 0.001
Median	32	41.50	44.50	
SD	9.60	10.01	11.48	
Range	20-45	23-51	19-51	
**MAS (0–4)**				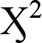 (2) = 0.87
Mean	0.58	0.63	0.65	*p* = 0.649
Median	0.56	0.64	0.61	
SD	0.28	0.35	0.43	
Range	0.22–0.94	0–1.11	0–1.28	
**WMFT (task rate)**^a^				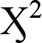 (2) = 6.20^*^
Mean	13.50	17.26	19.22	*p* = 0.045
Median	13.42	17.47	18.07	
SD	6.26	7.39	8.04	
Range	6.08–21.87	5.29–26.40	5.65–32.64	
**MAL-AOU (0–5)**				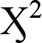 (2) = 15.20^***^
Mean	1.17	2.01	1.90	*p* = 0.001
Median	1.00	1.98	2.10	
SD	0.72	0.86	0.91	
Range	0.52–2.45	0.79–3.59	0.71–3.21	
**MAL-HW (0–5)**				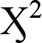 (2) = 15.00^***^
Mean	1.20	2.05	1.98	*p* = 0.001
Median	0.98	1.99	2.20	
SD	0.62	0.78	0.80	
Range	0.53–1.93	0.79–3.22	0.71–2.89	
**CAHM (0–100)**				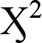 (2) = 5.40
Mean	45.83	62.01	58.39	*p* = 0.067
Median	44.50	58.38	53.25	
SD	16.92	21.26	18.70	
Range	26–80.75	28.50–98.55	30–86.50	
**SIS-Hand (0–100)**^b^				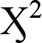 (2) = 11.74^**^
Mean	24	36.40	33.20	*p* = 0.003
Median	24	32	36	
SD	15.89	18.13	18.09	
Range	4–48	12–72	8–56	
**SIS-recovery (0–100)**				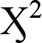 (2) = 4.67
Mean	62.80	69.50	71.30	*p* = 0.097
Median	60	70	72.50	
SD	12.07	14.42	12.61	
Range	50–87	40–85	50–88	

a *Task Rate indicates average # of times each test item could be completed within 1 min. Higher scores indicate improved task completion*.

b *SIS-Hand, Transformed scores reported [0–100]*.

* *p < 0.05*,

** *p < 0.01*,

*** *p < 0.001*.

Wilcoxon *post-hoc* tests of participant ratings on the Confidence in Arm and Hand Movement (CAHM) scale indicated that confidence in use of the paretic UE for a variety of functional activities (e.g., cutting food with a knife and fork or performing tasks in public) trended upward at the one-month follow-up visit, with admission to follow-up results reaching statistical significance (*p* = 0.037). Moderate to large Cohen's *d* effect sizes for these measures are reported in Table 5.

**Table 5:** Cohen's *d* effect sizes (*n* = 10).

**Outcome**	**Admission to discharge**	**Admission to follow-up**
Fugl-Meyer ASSESSMENT (FMA-UE)	*d* = 0.74	*d* = 0.69
WMFT (task rate)	*d* = 1.06	*d* = 0.89
Motor Activity Log (MAL-AOU)	*d* = 1.21	*d* = 1.09
Motor Activity Log (MAL-HW)	*d* = 0.85	*d* = 0.71
Confidence in Arm & Hand Movement (CAHM)	*d* = 0.56	*d* = 0.81
Stroke Impact Scale, Hand (SIS-Hand)	*d* = 0.73	*d* = 0.54

## Discussion

The clinical acceptance and widespread use of rehabilitation robots for UE therapy post-stroke has been limited, in part, by the lack of empirical evidence for its impact on UE performance and engagement in meaningful activities of daily living (4, 5). This development of concept pilot trial (25) is the first to test an ALPS that shifts robot-assisted therapy away from an impairment focused intervention to one aimed to facilitate the transfer of robot-trained motor skills to functional use of the paretic arm and hand after stroke. This new paradigm is based upon principles of experience-dependent neuroplasticity (7) and cognitive strategy training (15), and embraces the distinct strengths of robot-assisted technology and clinician-driven interventions. The rehabilitation robots deliver a higher dose of repetitive task-specific training than is possible in conventional rehabilitation settings, while the clinician empowers participants with a step-by-step problem-solving approach to facilitate use of trained motor skills during meaningful everyday activities, thereby adding salience and transference to the rehabilitation process.

The Mann Whitney U group analyses revealed statistically and clinically significant improvements in motor capacity, as measured by the FMA-UE, with the ALPS + RT group improving more than those who received a combination of ALPS + RT-TOT. Participants in the ALPS + RT group received on average a total of 524.0 min of Armeo®Spring and Amadeo™ training during the study protocol, as compared to 303.0 min in the ALPS + RT-TOT group. Although individuals randomized to the RT-TOT group also received repetitive task-oriented training during 20–30 min of each treatment session, it was not possible to achieve as many movement repetitions during this time due to the nature of the training, which was focused on guided discovery and problem solving during challenging, yet achievable UE tasks. The number of repetitions, choice of discrete vs. continuous tasks (e.g., reaching vs. stirring), and practice of unilateral and bilateral tasks during task-oriented training was individualized, based on the participant's UE motor capacity and target of intervention. Therefore, it is likely that individuals in the ALPS + RT group completed more movement repetitions than those in the RT-TOT group, which may have contributed to greater improvement in UE motor capacity, as measured by the FMA-UE.

Whyte et al. (26, 27) have developed the Rehabilitation Treatment Specification System to specify and study the effects of rehabilitation treatments and uncover the “black box” of rehabilitation. This framework is useful for describing the treatment outcomes or *targets* as well as the many treatment *ingredients* that comprise a given intervention and their potential *mechanisms of action*. The primary *target* for most robot-assisted therapy studies has been a reduction in motor impairment, with less attention to measuring gains in functional use of the paretic arm and hand during everyday activities. A missing element in much of this research is the examination of what treatment *ingredients* other than the number of repetitions delivered (e.g., type of human machine interface, instructions, motor skills practiced by robot therapy games) are integral to the intervention protocol, and how they contribute to changes in performance. An intervention study that compared effects of Amadeo™ robot-assisted therapy to conventional hand training by an occupational therapist revealed significantly greater improvements on neurophysiological measures of cortical plasticity and interhemispheric inhibition in the Amadeo™ group that paralleled gains in clinical outcome scores (28). Controlled studies such as this are essential to our understanding of the relationship between treatment ingredients delivered by these different forms of hand training and potential mechanisms of action that contribute to observed changes on standardized clinical assessments and in functional use of the paretic arm and hand after stroke.

The recently published RATULS randomized control study of more than 700 stroke participants who received robot-assisted therapy, enhanced upper limb training (EULT) by a rehabilitation clinician, or usual care reported that the intensive training interventions (robot- therapy and EULT) did not significantly improve its targeted outcome, UE function as measured by Action Research Arm Test (ARAT) (29). In addition, the small gains that were observed in UE function did not transfer to activities of daily living. These findings, and similar reports from systematic reviews of robot-assisted therapy (4, 5), indicate that greater attention is warranted to treatment ingredients other than repetition. While rehabilitation robots are highly capable of repetitive movement training, it is apparent that robot-assisted therapy alone is not sufficient for optimizing UE activity engagement and participation in persons with UE motor impairments after stroke. In the current ALPS protocol, treatment ingredients to specifically enhance the transfer of robot-trained motor skills included instruction in cognitive strategies to enhance problem solving during UE activities and a HAP to encourage carry-over of robot-trained motor skills to daily activities in the home and community. While the ALPS pilot was not designed to differentiate the effects of these treatment ingredients, the statistically significant gains and medium to large effect sizes for outcomes across ICF domains, coupled with clinically significant improvements in FMA-UE scores at follow up (n = 10, mean = 7.3/66 points) are promising. They far exceed gains reported in the 36 session RATULS study (adjusted mean FMA-UE difference of 2.79/66 points between robot and usual care groups at 3 months) and in systematic reviews of robot-therapy outcomes (5, 29). The present findings align with assertions by Valero-Cuevas et al. (30) that changes in performance are multidimensional and cannot be measured by a single primary outcome, such as the Fugl-Meyer Assessment or ARAT.

A systematic review of UE rehabilitation methods after stroke (31) emphasized the importance of tailoring evidence-based treatments to the needs of the individual. Each component of the ALPS protocol (robot therapy, cognitive strategies, and HAP) was individualized, based on the participant's level of UE functioning and identified task goals. The HAPs provided to ALPS participants were tailored to their individual interests and contexts, and were based upon prior research on the effectiveness of cognitive strategy training for individuals post-stroke (10, 32). While adherence to daily HAP completion varied among participants, semi-structured interviews administered more than 6 months post-ALPS training revealed that the HAPs were a separate, yet valued ingredient of the intervention. Participants applied ALPS strategies (e.g., STOP, THINK, DO, CHECK) to problem-solve challenges encountered during everyday tasks. Those with greater distal function at baseline were more likely to follow through with HAP activities for the paretic arm and hand and reported greater ability to independently apply problem solving strategies during HAP activities. Participants who did not consistently complete HAP activities suggested ways to improve adherence, including discussions to better manage fatigue, time management, and potential benefits of a computer or mobile application to improve ease of reporting. Thematic analysis of post-intervention interviews has begun, and the initial results have contributed to our understanding of the treatment ingredients most beneficial to past participants. Many reported continued use of the ALPS strategies more than 1-year post-intervention and viewed each treatment component as essential to improving use of their paretic arm and hand during daily activities. Participant input has been used to refine the intervention manual prepared for our next ALPS trial.

Limitations of this research, including its small sample size and variable daily adherence to the HAP across participants, suggest caution when interpreting study outcomes. The inclusion criteria limited our participant sample to individuals with moderate upper extremity impairments as measured by the Fugl-Meyer Assessment (inclusion range 21–50/66 points), therefore generalization of findings to individuals with milder or more severe impairments is limited. Also, our participants were individuals more than 6 months post-stroke onset, and many had developed learned non-use of the paretic arm and hand during this time. Earlier training and implementation of ALPS strategies during acute and subacute phases of recovery may facilitate greater ease of transfer and adherence to HAP activities.

## Conclusions

The novel Active Learning Protocol for Stroke (ALPS) has the potential to shift current research paradigms for intensive robot-assisted therapy by training stroke participants to engage in self-analysis and active problem solving to better utilize recovered UE motor skills during daily living tasks. This innovative project is the first to extend this cognitive strategy and motor learning approach to robot-assisted therapy for persons with moderate UE impairments after stroke: individuals who may not qualify for task-oriented training protocols. The ALPS protocol and client-centered HAP are derived from principles of experience-dependent neuroplasticity (7), motor learning strategies applied to task-oriented training (8, 12) and the Cognitive Orientation to daily Occupational Performance (15). Although this initial pilot study to develop and test the ALPS protocol was well-tolerated and produced significant gains in paretic UE capacity and performance, we are in the process of refining and formalizing the intervention protocol in preparation for a larger confirmatory trial.

## Data Availability Statement

The datasets generated for this study are available on request to the corresponding author.

## Ethics Statement

The studies involving human participants were reviewed and approved by Partners Human Research Committee (PHRC), the IRB for Partners Healthcare in Boston, MA. The patients/participants provided their written informed consent to participate in this study. Written informed consent was obtained from the individual(s) for the publication of any potentially identifiable images or data included in this article.

## Author Contributions

SF developed the Active Learning Program for Stroke research protocol, obtained IRB approval, administered the intervention, and was primarily responsible for data analysis, interpretation, and writing of the manuscript. CA-D was a blinded evaluator, assisted with data analysis, interpretation of results, and editing of the manuscript.

### Conflict of Interest

SF consulted with the manufacturer of the Amadeo robot (Tyromotion, Graz AT) on the rehabilitation potential of another robot in development. Tyromotion has loaned the Amadeo robot used for this research to the MGH Institute of Health Professions. The remaining author declares that the research was conducted in the absence of any commercial or financial relationships that could be construed as a potential conflict of interest.

## Abbreviations

ALPS: Active Learning Program for Stroke
CO-OP: Cognitive Orientation to daily Occupational Performance
CAHM: Confidence in Arm & Hand Movement
FMA-UE: Fugl-Meyer Assessment—upper limb subtests
HAP: Home Action Plan
MAS: Modified Ashworth Scale
MAL: Motor Activity Log
RT: Robot-assisted Therapy
SIS: Stroke Impact Scale
TOT: Task-Oriented Training
WMFT: Wolf Motor Function Test.
