# Supplementary material for: A Paradigm Shift: Rehabilitation Robotics, Cognitive Skills Training, and Function After Stroke
Source: Front Neurol. 2019 Oct 15;10:1088. doi: 10.3389/fneur.2019.01088 (PMC6804158; doi:10.3389/fneur.2019.01088)
Supplement: Supplementary file 1 [file Data_Sheet_1.docx]

**Appendix A. Active Learning Program for Stroke (ALPS) Strategies.**

| **ALPS General Strategies – applicable to all tasks and desired activities** | |
| --- | --- |
| **Stop** | - What are you trying to accomplish? - Notice what part of the activity or movement is especially challenging for you.   - Are you having a problem with reaching?   - Controlling movement at multiple joints at the same time?   - Positioning your forearm or wrist before grasping an object?   - Using your hand to hold or manipulate an object? |
| **Think** | - Which adaptive strategy might be useful for this challenge? - Select a strategy based on what you think is the most challenging part of the task (see next page!) |
| **Do** | - Try your strategy, paying attention to what part of the activity or movement was challenging you. |
| **Check** | - Is your performance improved by the strategy you tried?   - If yes – how might you use this strategy for a similar task?   - If no – why not? Was the strategy partly helpful?     - If yes, try a new strategy.     - If faced with a new challenge, what alternative approach or strategy might be useful (go back to THINK stage!) |
| **ALPS Movement Strategies** | |
| If you are having difficulty with reaching from the shoulder, DO:   - Stretch to relieve excess joint tightness, increased tone. - Assist at the elbow during reach by helping with your other hand. - Grade the distance you need to reach – try reaching to targets at or below waist level, and gradually increase how high or far you reach as you improve. - Other: | |
| If you are having difficulty with controlling movement at multiple joints at the same time, DO:   - Reduce the degrees of freedom by providing assist proximal to the motion you are trying to accomplish   - For example, stabilize at the forearm if trying to control the wrist and fingers for grasping an object. - If sitting, rest your elbow on the table surface as you try to control elbow, forearm, wrist or finger movements (this will reduce control you need at the shoulder!). - Try to identify which part of the movement or task is most challenging, ask for help with problem solving! - Other: | |
| If you are having difficulty with positioning your forearm or wrist before grasping an object, DO:   - Try the activity with your less affected arm and hand – look at how you position your forearm or wrist before grasp.   - Try to mimic this movement with your weaker arm/hand.   - If unable to mimic the movement, help to position your forearm or wrist with your other hand. - Pay attention to the position of your forearm and/or wrist – can you position your forearm & wrist first, then try to grasp or manipulate an object? - Other: | |
| If you are having difficulty with using your hand to hold or manipulate an object, DO:   - Check the position of your forearm and wrist (see above!). - Focus on maintaining your grasp as you move your arm to reach. - Weight bear or stretch your arm and hand if tight. - Adapt the activity.   - Use larger handled utensil.   - Use your weaker arm/hand to hold vs. manipulate. - Other: | |
